# Classifying breast cancer subtypes on multi-omics data via sparse canonical correlation analysis and deep learning

**DOI:** 10.1186/s12859-024-05749-y

**Published:** 2024-03-27

**Authors:** Yiran Huang, Pingfan Zeng, Cheng Zhong

**Affiliations:** 1 School of Computer and Electronics Information, Guangxi University, Nanning, 530004 China; 2 Guangxi Key Laboratory of Multimedia Communications Network Technology, Nanning, 530004 China

**Keywords:** Multi-omics data integration, Breast cancer subtypes, Sparse canonical correlation analysis, Deep neural network

## Abstract

**Background:**

Classifying breast cancer subtypes is crucial for clinical diagnosis and treatment. However, the early symptoms of breast cancer may not be apparent. Rapid advances in high-throughput sequencing technology have led to generating large number of multi-omics biological data. Leveraging and integrating the available multi-omics data can effectively enhance the accuracy of identifying breast cancer subtypes. However, few efforts focus on identifying the associations of different omics data to predict the breast cancer subtypes.

**Results:**

In this paper, we propose a differential sparse canonical correlation analysis network (DSCCN) for classifying the breast cancer subtypes. DSCCN performs differential analysis on multi-omics expression data to identify differentially expressed (DE) genes and adopts sparse canonical correlation analysis (SCCA) to mine highly correlated features between multi-omics DE-genes. Meanwhile, DSCCN uses multi-task deep learning neural network separately to train the correlated DE-genes to predict breast cancer subtypes, which spontaneously tackle the data heterogeneity problem in integrating multi-omics data.

**Conclusions:**

The experimental results show that by mining the associations among multi-omics data, DSCCN is more capable of accurately classifying breast cancer subtypes than the existing methods.

## Introduction

Breast cancer is the second leading cause of cancer death in women after Lung cancer [1]. It is a highly heterogeneous disease, consisting of different biological subtypes. Each breast cancer subtype has different clinical, pathological and molecular features, and has different prognostic and therapeutic implications [2, 3]. Therefore, the study of breast cancer subtypes is of great significance for precision medicine and prognosis prediction of breast cancer [4, 5]. To profile heterogeneous genotype data related to breast cancer, high-throughput technologies could be exploited [6–8].

Driven by the new high-throughput sequencing technologies, biological data in a variety of different formats, sizes and structures are growing at an unprecedented rate [9–11]. Based on these omics data, there have been many studies on the classification of breast cancer subtypes, which can be divided into two categories. The first category is based on single omics data. For example, Lehmann et al. [12] used gene expression data for clustering analysis to identify subtypes of triple-negative breast cancer. Rhee et al. [13] proposed a hybrid approach to integrate graph convolutional networks and relational networks to predict breast cancer subtypes using gene expression profiles. Yu et al. [14] performed differential expression analysis on biologically important genes in the gene regulatory networks and constructed a machine learning-based binary classification model for each breast cancer subtype using the differential expression genes. Each type of omics data exhibits specific disease associations [15, 16]. However, the analysis of single omics data do not capture the interrelationships between molecules at different levels, which may fail to provide a comprehensive understanding of the biological processes of breast cancer [17].

To address these limitations, the second category utilizes multi-omics data to perform breast cancer classification. Various studies have shown that combining multiple omics datasets yields better accurate prediction to clinical outcomes, thereby verifying the importance of integrating multi-omics data over single-omics data [17–21]. According to the way of data integration, the multi-omics data integration methods for predicting breast cancer subtypes can be classified as concatenation-based, ensemble-based and knowledge-driven methods[22].

The concatenation-based methods combine all omics data into a single dataset before training [15, 23]. For example, Tao et al. [24] presented a SVM model with multiple kernel to classify breast cancer subtypes using multi-omics data. List et al. [25] constructed random forest model to classify breast cancer subtypes using both gene expression and DNA methylation data. Concatenation-based methods are convenient for integrating multi-omics data into single dataset before training, but they suffer from the increasing dimensionality of multi-omics data and the data heterogeneity issue in integrating multi-omics data [26]. The ensemble-based methods separately train a model on each omics dataset and combine the prediction results based on the average or majority voting scheme [27]. For example, Lin et al. [28] proposed a deep neural network model DeepMo based on multi-omics data for the breast cancer subtypes classification. DeepMo applies fully-connected layers to each omics and concatenates these fully-connected layers for final subtypes prediction. Joung et al. [29] presented an interpretable deep learning-based framework moBRCA-net for classifying breast cancer subtypes. moBRCA-net utilizes self-attention module to each omics to mine the important features of multi-omics data and integrates the mined features into deep neural network to identify breast cancer subtypes. The ensemble-based methods retain unique data distribution so that the omics data from different sources can be fully trained. However, the ensemble-based methods do not consider the biological interaction between multi-omics data, which may lose complementary information in multi-omics data [30]. Knowledge-driven approaches considers the relationships between different omics data based on prior knowledge. For example, Singh et al. proposed DIABLO to seek common information across different modality data by selecting a subset of features and discriminating multiple subtypes simultaneously. SMSPL [31] is a robust multimodal approach for classifying breast cancer subtypes by analyzing integrative multi-omics data. However, it should be noted that the prior knowledge sometimes may not be suitable for some biological research fields [31].

Although the abovementioned methods have achieved great success in predicting breast cancer subtypes, some challenges still remain when integrating multi-omics data: (1) Biological data usually contain a large number of features *p* and small size of samples *n*, which is called the large *p* and small *n* problem [32]. From a biological perspective, only a small fraction of features is highly correlated with the target disease, while most features are irrelevant. From a machine learning perspective, many irrelevant features may be prone to overfitting problems and negatively influence the performance of the classifier. (2) Data heterogeneity problem. Different types of biological data produced by different omics platforms contain heterogeneous information, which could result in different kinds and levels of uncertainty and imprecision [33]. (3) The complementary information presented in multi-omics data is not fully utilized. In the classification of breast cancer subtypes, people mainly focused on employing the associations between disease and single omics data rather than the associations among different types of omics data.

Motivated by these limitations, we propose a novel framework called DSCCN for classifying breast cancer subtypes by mining the associations among multi-omics data. To solve the large *p* and small *n* problem in the integration of multi-omics data, DSCCN first performs differential analysis on the multi-omics expression data of breast cancer patients to identify differentially expressed genes. This step, specifically designed for breast cancer, has effectively reduced the number of features while ensuring that the selected features are statistically significant, which are potentially related to the occurrence of breast cancer. To mine the associations among multi-omics data, a SCCA mode [34] is exploited to detect linear structural interaction information of the multi-omics expression data to uncover correlated multi-omics features of the identified DE-genes. To the best of our knowledge, this is the first time of using SCCA model to identify associations in multi-omics data for classifying breast cancer subtypes. Finally, DSCCN adopts an end-to-end multi-task deep learning neural network model DNN with attention mechanism to train the correlated multi-omics features of DE-genes to classify the breast cancer subtypes. Unlike traditional neural networks, which are usually trained only for a single specific task, our multi-task network utilizes a shared representation to perform multiple tasks simultaneously. Two independent tasks are separately performed to train our DNN model on two omics dataset, and the attention mechanism is utilized to mine the impotrant multi-omics genes of high similarity within both tasks to produce classification probabilities for each task. This effectively solves the problem of data heterogeneity and captures the information presented in multi-omics.

We demonstrate the capability of DSCCN by comparing it with the state-of-the-art methods. In the comparative experiments, we evaluate the performance of all competitive methods in the binary/multiclass classification of breast cancer subtypes. The results demonstrate that DSCCN shows competitive performance with the existing methods in classifying breast cancer subtypes. Our proposed DSCCN thus could be a promising method for the classification of breast cancer subtypes. The source code is available at https://github.com/hyr0771/DSCCN.

## Materials and methods

**Fig. 1:**
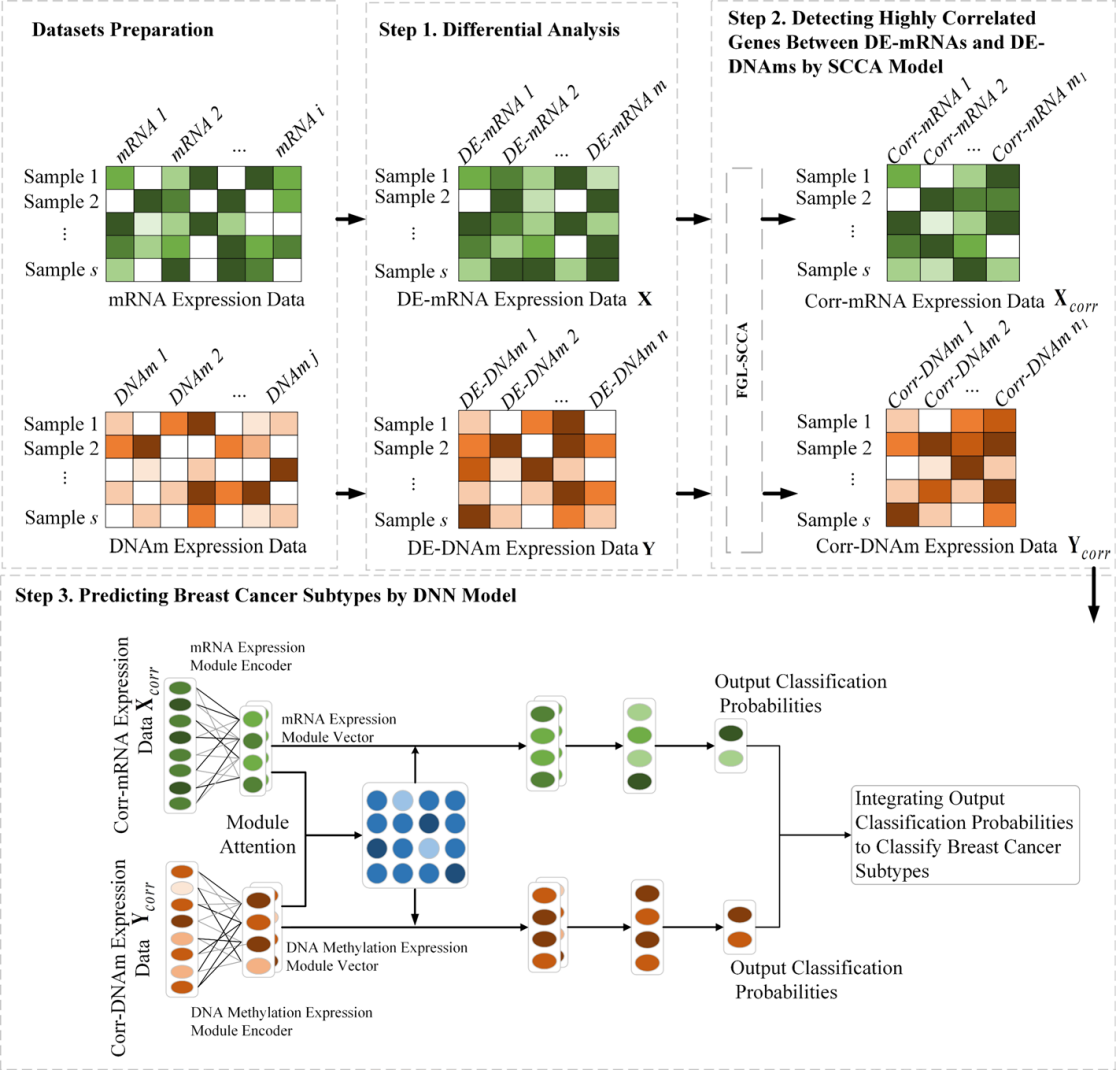
Procedure of DSCCN. Step 1 first performs differential analysis on mRNA and DNA methylation (DNAm) omics data to find DE-genes. Step 2 uses a SCCA model to detect highly correlated genes in mRNA and DNAm using DE-genes. Step 3 utilizes correlated genes to train the deep neural network model DNN to classify the breast cancer subtypes

In this section, we introduce our method DSCCN for classifying breast cancer subtypes. The overview of DSCCN is summarized in Fig. 1. As shown in Fig. 1, DSCCN mainly includes three steps:

- Step 1: Performing differential analysis on the multi-omics data (mRNA, DNA methylation) of breast cancer patients to detect DE-mRNAs and DE-DNAms.
- Step 2: Utilizing Sparse Canonical Correlation Analysis to identify highly correlated mRNAs and DNAms of patients based on the detected DE-mRNAs and DE-DNAms in step 1. We call these correlated mRNAs and DNAms as Corr-mRNAs and Corr-DNAms, respectively.
- Step 3: Using the deep neural network model to classify the breast cancer subtypes based on the Corr-mRNAs and Corr-DNAms of patients.

### Differential analysis of multi-omics data

The breast cancer multi-omics (mRNA, DNAm) data of patients are obtained from The Cancer Genome Atlas(TCGA) [35]. The multi-omics data contains four subtypes of breast cancer: Basal-like (Basal), Her2-enriched (Her2), Luminal A (LumA), Luminal B (LumB), which are publicly reported as the most replicated subtypes of human breast cancer [2]. The primary characteristics of the breast cancer subtypes are based on the expression levels of estrogen receptor (ER), progesterone receptor (PR), human epidermal growth factor receptor 2 (HER2) and proliferation indicator Ki67 [2, 36, 37]. The sample numbers of the breast cancer subtypes are given in Table 1.

**Table 1:** The original and differential analysis mRNA and DNA methylation data

Omics types	Original data		Differential analysis data	
	Sample	No. of features	Sample	No. of features
mRNA	1180	19,961	528	3692
DNA methylation	882	12,264	528	4679

In the entire sample set (528), the distribution of breast cancer subtypes is: Basal (87), Her2 (31), LumA (284), and LumB (126)

Note that integrating omics data faces the challenge of the large *p* and small *n* problem. Appropriate dimensionality reduction is necessary for identifying relevant multi-omics features of samples. We thus first carry out dimensionality reduction process on the mRNA and DNAm datasets. Specifically, we divide the samples into two groups. For the mRNA dataset, the health group and the disease group with breast cancer contain 194 and 986 samples respectively. For the DNAm dataset, the health group and the disease group with breast cancer contain 97 and 785 samples respectively.

**Fig. 2:**
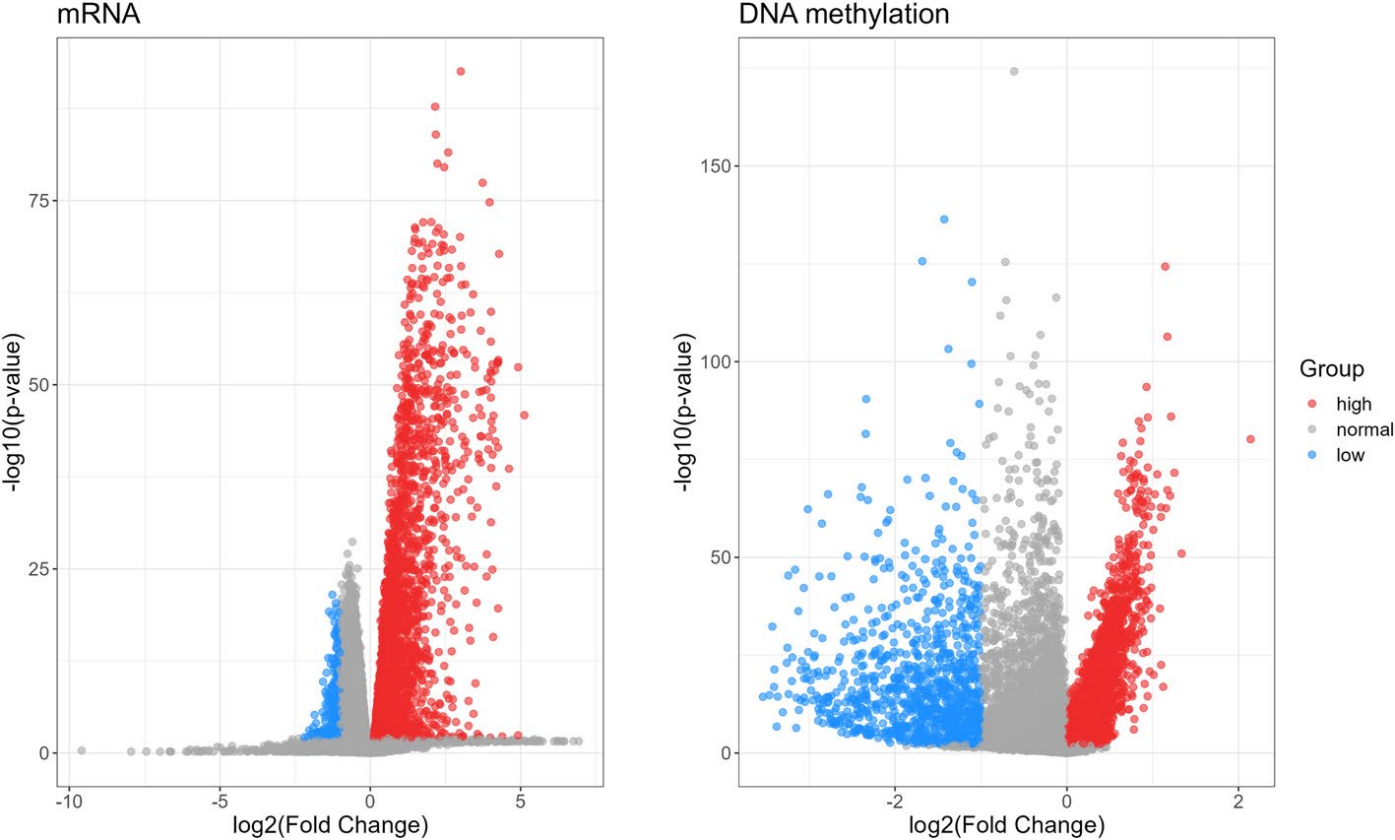
Volcano plots of differentially expressed mRNAs and DNAms between the health and disease groups of breast cancer patients. Red dots and blue dots represent the highly and lowly expressed genes, respectively. Grey dots represent normal expressed genes. The highly and lowly expressed genes are considered as differentially expressed genes (DE-genes)

Then we perform differential analysis on two sets of omics separately, utilizing T-test and Fold Change methods to identifying differentially expressed genes. Specifically, the genes with a *p*-value (T-test) less than 0.01 and a Fold Change less than 0.5 are defined as lowly expressed genes. Similarly, those with a *p*-value (T-test) less than 0.01 and a Fold Change greater than 1 are considered highly expressed genes. Finally, we totally obtain 3692 DE-mRNA genes, with 3440 highly expressed genes and 252 lowly expressed genes; 4679 DE-DNAm genes, with 3740 highly expressed genes and 939 lowly expressed genes. The results of differential analysis of the mRNA and DNAm data are shown in Table 1 and Fig. 2.

### Identifying correlated genes with SCCA model

A comprehensive analysis of mRNA and DNA methylation omics data can offer a encompassing overview of gene regulation, aiding in the comprehension of the molecular mechanisms for gene expression regulation. Detecting complex bi-multivariate associations between the mRNA and DNAm of patients is a critical task in identifying cancer subtypes. Recently, Sparse Canonical Correlation Analysis has received great attention in bi-multivariate association identification and feature selection [34]. Usually, there exists a chain association across mRNA and DNAm [38, 39]. Specifically, the effect of DNA methylation on mRNA is mainly manifested in its ability to regulate gene expression changes in DNA methylation levels can affect the binding of transcription factors to DNA, leading to activation or silencing of genes, which in turn affects the production of mRNA. Inspired by this, we adopt a SCCA model called FGL-SCCA [34] with the fused pairwise group lasso (FGL) penalty and the graph guided pairwise group lasso (GGL) penalty to mine the bi-multivariate associations of mRNA and DNAm to classify breast cancer subtypes.

The matrix $\mathbf{X}\in {\mathcal{R}}^{s\times m}$ represents the DE-mRNA data of patients where *s* is the number of samples and *m* is the feature number of DE-mRNA. The matrix $\mathbf{Y}\in {\mathcal{R}}^{s\times n}$ represents the DE-DNAm data of patients where *n* is the feature number of DNAm. Let $\mathbf{X}$ and $\mathbf{Y}$ be normalized and centered, the optimization problem can be defined as the following FGL-SCCA model [34]:

1 $$\mathop{\mathbf{min}}\limits_{\mathbf{u},\mathbf{v}} -{\mathbf{u}}^{{\top }}{\mathbf{X}}^{{\top }}\mathbf{Yv}+{\varphi }_{FGL}\left(\mathbf{u}\right)+{\varphi }_{GGL}\left(\mathbf{v}\right)\text{ s.t. }\parallel \mathbf{Xu}{\parallel }^{2}\le 1,\parallel \mathbf{Yv}{\parallel }^{2}\le 1,$$

where the vectors **u** and **v** are the canonical weights for the mRNA features and DNAm features respectively, ${\varphi }_{FGL}(\mathbf{u})$ and ${\varphi }_{GGL}(\mathbf{v})$ are the penalties to fit the adjacent smoothness and graphical smoothness, respectively. The FGL penalty ${\varphi }_{FGL}(\mathbf{u})$ is defined as ${\gamma }_{1}\sum_{k=1}^{m-1} {\omega }_{k,k+1}\sqrt{{u}_{k}^{2}+{u}_{k+1}^{2}}$ where ${\omega }_{k,k+1}$ is the weight of two adjacent features and ${\gamma }_{1}$ is positive tuning hyperparameter. By mapping the feature space of $\mathbf{v}$ into a undirected graph *G*, the GGL penalty ${\varphi }_{GGL}(\mathbf{v})$ is defined as ${\gamma }_{2}\sum_{\left(p,q\right)\in E} {\omega }_{p,q}\sqrt{{v}_{p}^{2}+{v}_{q}^{2}}$ where *p* and *q* are the DNAm feature nodes of *G*, *E* is the edge set guided by the graph *G*, and ${\omega }_{p,q}$ is the weight of the edge, and ${\gamma }_{2}$ is a hyperparameter to control the amount of regularization. Both FGL and GGL penalty can be used in the data-driven model in the case of no prior knowledge is given[34], while FGL assumes that the mRNA data is sequential. Meanwhile GGL is usaully adopted to bridge the gap between graph guided penalties and group lasso. DNA methylation have different roles in cell types or tissues and the graphical relationship of different roles for DNA methylation could be better captured by the graph guided penalty GGL. We thus impose the FGL penalty on mRNA data and GGL penalty on DNA methylation data, respectively.

The FGL penalty encourages ${u}_{k}$ and ${u}_{k+1}$ in the vector $\mathbf{u}$ to have similar values. During each iteration of solving Eq. (1), the FGL penalty sets ${\omega }_{k, k+1}$ to the value of ${u}_{k}^{2}$ in the previous iteration. This forms a smooth sequence of weights among adjacent elements of $\mathbf{u}$, which is beneficial for handling data with an ordered structure.

For the GGL penalty imposing on $\mathbf{Y}$, the undirected graph *G* represents the pattern of connections between the DNAm features, guiding the construction of the edge set *E*. Specifically, a matrix of *n* $\times$(*n*-1) rows and *n* columns is constructed for *E* where each row represents a connection between two different nodes in *G*. For the connected DNAm feature nodes *p* and *q* in *G* and their canonical weights ${v}_{p}$ and ${v}_{q}$, the GGL penalty encourages ${v}_{p}$ and ${v}_{q}$ in $\mathbf{v}$ to have similar values. Similar to the determination of ${\omega }_{k, k+1}$, the GGL penalty sets ${\omega }_{p,q}$ to the value of ${v}_{p}^{2}$ in the previous iteration of solving Eq. (1).

Based on the DE-mRNA features and DE-DNAm features derived from the differential analysis in the first step, we adopt standard quadratic programming [34, 40] to solve (1), and the solutions $\mathbf{u}$ and $\mathbf{v}$ are the canonical weights for the DE-mRNA features and DE-DNAm features respectively. Then we can compute the correlation coefficient ${\text{corr}}(\mathbf{X*u},\mathbf{Y*v})$ to measure the relevance of the DE-mRNA and DE-DNAm features based on Pearson correlation coefficient. The larger the absolute value of the correlation coefficient, the stronger the correlation between the DE-mRNA features and DE-DNAm features. We can choose suitable values of ${\gamma }_{1}$ and ${\gamma }_{2}$ based on the correlation coefficient.

Finally, we calculate the absolute values of $\mathbf{u}$ and $\mathbf{v}$ and sort the DE-mRNA features and DE-DNAm features based on the values of $\mathbf{u}$ and $\mathbf{v}$ in descending order. Then we select the top ${m}_{1}$ DE-mRNA and ${n}_{1}$ DE-DNAm features to construct the correlation matrices ${\mathbf{X}}_{corr}\in {\mathcal{R}}^{s\times {m}_{1}}$ and ${\mathbf{Y}}_{corr}{\in \mathcal{R}}^{s\times {n}_{1}}$.

### Predicting breast cancer subtypes using DNN model

The FGL-SCCA model is capable of extracting linear structured feature information from the mRNA and DNAm data. However, the non-linear associations in the omics data are critical for cancer subtype classification as well. In order to mine the non-linear associations in the mRNA and DNAm data, we utilize a multi-task deep learning neural network model DNN [41] to identify the non-linear associations among the mRNA and DNAm data to predict breast cancer subtypes. We use ${\mathbf{X}}_{corr}$ and ${\mathbf{Y}}_{corr}$ as the input of the DNN model. As can be seen in Fig. 1, the DNN model consists of three main stages: (i) constructing modules for each dataset using module encoder. (ii) Identifying important modules across different omics data with a module attention mechanism. (iii) Implementing multi-task learning on a fully connected layer to comprehensively process each omics dataset.

#### Module encoder

The module encoder consists of a fully connected layer, which links the features of the omics data to each module. Let ${\mathcal{W}}_{\text{module }}^{j}$ denote the weights of the fully connected layer, which represents the association between modules and features of the *j-th* omics data. For a training sample (*x *^*j*^,* y*), *x *^*j*^ denotes the sample from the *j*-*th* omics data and *y* is the classification label of* x *^*j*^. Let ${\mathcal{F}}_{module}^{j}$ represent the module encoder for the *j-th* omics data. The module vectors ${M}^{j}$ for the *j-th* omics data can be defined as follows:

2 $${M}^{j}\left({x}^{j}\right)={\mathcal{F}}_{\text{module }}^{j}\left({x}^{j};{\mathcal{W}}_{\text{module }}^{j}\right)\in {\mathcal{R}}^{{N}^{j}\times D},$$

where ${\mathcal{W}}_{\text{module}}$ represents the weights of ${\mathcal{F}}_{\text{module}}$, ${N}^{j}$ indicates the number of modules of *j*-*th* omics data, and $D$ represents the dimension of the module vector.

#### Attention mechanism

DNN devises a module attention mechanism that specifically focus on modules with high similarity between each omics data module. Cosine similarity is used to assess the degree of correlation among these modules. Let $Att$ denote the module attention matrix between the module vectors of two omics datasets. ${Att}_{lk}$ represents the element in row* l* and column *k* of $Att$. ${Att}_{lk}$ contains the information on the potential dependencies between the *l-th* module of one omics dataset and the *k-th* module from another omics dataset. The definition of each element within the attention matrix is as follows [41]:

3 $$\begin{aligned} & Att_{{lk}} \left( {M^{i} ,M^{j} } \right) = \frac{{{\text{exp}}\left( {{\text{cos}}\left( {M_{l}^{i} ,M_{k}^{j} } \right)} \right)}}{{\sum _{{k = 1}}^{{N^{j} }} {\text{exp}}\left( {{\text{cos}}\left( {M_{l}^{i} ,M_{k}^{j} } \right)} \right)}} \\ & {\text{s.t.}}\;i,j \in 1, \ldots ,J,i \ne j, \\ \end{aligned}$$

where ${M}^{j} ={M}^{j}\left({x}^{j}\right)$ as an abbreviation, ${M}_{l}^{i}$ and ${M}_{k}^{j}$ respectively represent the *l*-th module vector of *i*-th omics data and the *k*-th module vector of *j*-th omics data. To emphasize important modules, the module vectors are multiplied by the attention matrices and then concatenated with the other omics data. The updated module vector is defined as follows:

4 $${Att\_M}^{j}\left({x}^{j}\right)=\left[{\left(Att\left({M}^{j},\overline{{\text{M} }^{ {\text{j}}}}\right)\right)}^{T}{M}^{j}\right],\text{ s.t. }\overline{{\text{M} }^{ {\text{j}}}}\in \left\{M\mid M\ne {M}^{j}\right\}$$

#### Training

The fully connected layers are then applied. In the model, loss $\mathcal{L}$ is set to the cross-entropy error between the true label and predict outputs and it is defined as follows:

5 $$L = - \sum\limits_{j = 1}^{J} {\sum\limits_{i = 1}^{C} {y_{i} } } \cdot \log \left( {\hat{y}_{i} } \right),$$

where $J$ denotes the number of omics datasets, $C$ represents the total number of the breast cancer subtypes, $y_{i} { }\left( {\hat{y}_{i} } \right)$ denotes the true (predict) probability for each breast cancer subtype. Each layer takes the previous layer as input and multiplies it with the trained weight matrix to obtain the input of the next layer. At last, the classification layer flattens the multi-dimensional vectors and generates the final classification probabilities for each breast cancer subtype.

## Results

### Evaluation metrics

In this section, we will introduce the metrics for evaluating the performance of classifying breast cancer subtypes. The number of correctly predicted positive samples is denoted as *TP* (True Positive) and the number of negative samples that are identified as positive samples is denoted as *FP* (False Positive). Similarly, the number of correctly predicted negative samples is denoted as *TN* (True Negative), and the number of the positive samples that are identified as negative samples is denoted as *FN* (False Negative). Then we can calculate the Accuracy(ACC) = (*TP* + *TN*)*/*(*TP* + *TN* + *FP* + *FN*), Precision = *TP/*(*TP* + *FP*), Recall = *TP/*(*TP* + *FN*) and F1 = *2* × *Precision* × *Recall/*(*Precision* + *Recall*). Accuracy (ACC) indicates the prediction accuracy of all samples whereas Precision indicates the ratio of the true positive samples in the predicted positive samples. Recall indicates the probability that the true positive samples are correctly predicted. ROC is the curve that calculates True Positive Rate *TPR* = *TP/*(*TP* + *FN*) and False Positive Rate *FPR* = *FP/*(*TN* + *FP*) according to various rank thresholds. AUC is defined as the area under the ROC curve and it is less than 1.

Traditional metrics such as Precision, Recall, and F1 score are originally defined for binary classification problems. In multi-classification problems, we use macro-averaged Precision (Precision-macro), macro-averaged Recall (Recall-macro), and macro-averaged F1 score (F1-macro) to comprehensively evaluate the performance of each method. Specifically, we first independently calculate the Precision, Recall, and F1 score for each class, and then respectively take the arithmetic mean of the Precision, Recall and F1 score across all classes to obtain Precision-macro, Recall-macro and F1-macro.

### Comparison with other methods

To evaluate our proposed method DSCCN, we compare its performance with the state-of-the-art methods. Specifically, we apply the logistic regression model/multinomial model with Elastic Net (EN) regularization [42], Random Forest (RF) [43] in the concatenation and ensemble frameworks to obtain two concatenation-based methods (Concate EN, Concate RF) and two ensemble-based methods (Ensemble EN, Ensemble RF) for comparison. Besides these four comparative methods, we also compare the performance of DSCCN with other three breast cancer classification methods based on multi-omics data. These three multimodal methods include DIABLO [22], SMSPL [31] and DeepMO [28].

Among the comparative methods, DIABLO is dedicated to maximizing the shared or correlated information across multiple omics datasets, reducing the high dimensionality of features. SMSPL addresses the issue of data heterogeneity by interactively recommending high-confidence samples between different modalities and assigns varying weights to training samples through its unique soft weighting mechanism, which significantly mitigates the impact of high-dimensional noise on model performance. Meanwhile, DeepMo employs the Select*K*Best [44] method from the Python library to select the top *K* features for training to alleviate the problem of data heterogeneity.

In the experiments, we use FGL-SCCA to detect highly correlated genes between DE-mRNAs and DE-DNAms. We randomly divided 70% of the samples as the training set and treated the remaining samples as the test set in Table 1. By performing grid search on ${\gamma }_{1}$ and${\gamma }_{2}$, we obtained the optimal correlation coefficient values of 0.969 on the training data and 0.896 on the test data, respectively. For DNN, the optimized parameters are as follows: the ‘number of modules’ is selected from {16, 32, 64, 128}, the ‘learning rate’ is selected from {10^–4^, 10^–5^, 5 × 10^–6^, 10^–6^}, the ‘weight decay’ is selected from {10^–3^, 10^–4^, 10^–5^} and the ‘early stopping patience’ is selected from {50, 100, 200, 300}. To ensure fairness in comparison, for each comparative method, including random forest, we used the default parameter value suggested by their literatures.

In the following section, we first verify the performance of DSCCN on the binary and multiple classification of breast cancer subtypes. Then we conduct ablation studies to learn the effectiveness of each step in DSCCN. Finally, we perform comprehensive analysis on the selected genes to learn the ability of DSCCN in identifying critical features for predicting breast cancer subtypes.

#### Performance of binary classification

To assess the performance of our method DSCCN in binary classification, we compare its effectiveness in distinguishing any two subtypes of breast cancer, including (1) Basal versus Her2, (2) Basal versus LumA, (3) Basal versus LumB, (4) Her2 versus LumA, (5) Her2 versus LumB, and (6) LumA versus LumB. The sample size of the breast cancer datasets in binary classification can be found in Table 2.We maintain the stability of our results by conducting stratified fivefold cross-validation on each classification dataset, and repeat the experiments 30 times to report the average measurement. The Accuracy, AUC and F1 score on any two subtypes of breast cancer obtained by different methods are shown in Table 3.

**Table 2:** The sample size of the breast cancer datasets in binary classification

Binary classification datasets	Total number of samples
Basal (87) vs Her2 (31)	118
Basal (87) vs LumA (284)	371
Basal (87) vs LumB (126)	213
Her2 (31) vs LumA (284)	315
Her2 (31) vs LumB (126)	157
LumA (284) vs LumB (126)	410

**Table 3:** Performance of binary classification for the subtypes of breast cancer

Breast Cancer Subtypes	Ensemble RF	Ensemble EN	Concate RF	Concate EN	DIABLO	SMPSL	DeepMo	DSCCN
*Accuracy*								
Basal vs Her2	0.826	0.870	0.826	0.782	0.857	0.913	0.912	**0.926**
Basal vs LumA	0.946	0.959	0.946	0.919	0.911	0.959	0.941	**0.982**
Basal vs LumB	0.923	0.929	0.952	0.952	0.935	0.786	0.948	**0.965**
Her2 vs LumA	0.905	0.921	0.921	**0.952**	0.920	0.825	0.910	0.951
Her2 vs LumB	0.839	0.839	0.839	0.839	0.864	0.774	0.908	**0.926**
LumA vs LumB	0.756	0.732	0.829	0.841	0.814	0.732	0.783	**0.844**
*AUC*								
Basal vs Her2	0.795	0.921	0.779	0.772	0.812	0.909	**0.989**	0.982
Basal vs LumA	0.889	0.929	0.907	0.857	0.850	0.850	0.939	**0.997**
Basal vs LumB	0.921	0.929	0.952	0.950	0.811	0.735	0.978	**0.997**
Her2 vs LumA	0.625	0.688	0.643	0.700	0.620	0.775	**0.983**	0.948
Her2 vs LumB	0.643	0.723	0.662	0.770	0.536	0.755	0.938	**0.951**
LumA vs LumB	0.778	0.695	0.797	0.769	0.670	0.751	0.838	**0.857**
*F1 score*								
Basal vs Her2	0.875	0.875	0.875	0.828	0.761	0.929	0.914	**0.933**
Basal vs LumA	0.875	0.875	0.882	0.833	0.985	0.824	0.927	**0.988**
Basal vs LumB	0.914	0.914	0.950	0.947	0.987	0.640	0.957	**0.974**
Her2 vs LumA	0.400	0.400	0.444	0.571	0.983	0.560	0.837	**0.973**
Her2 vs LumB	0.444	0.444	0.444	0.667	0.914	0.667	0.917	**0.956**
LumA vs LumB	0.811	0.804	0.875	0.896	0.740	0.784	0.664	**0.883**

The best results are marked in bold

Table 3 presents the performance comparison, demonstrating that DSCCN consistently outperforms other methods in terms of F1 score across all datasets. Notably, except for Her2 vs LumA, DSCCN attains the highest accuracy (ACC) on the remaining five datasets. Moreover, DSCCN attains the highest AUC value in four out of the six datasets. These results indicate that DSCCN is an effective method in performing binary classification for the subtypes of breast cancer.

#### Performance of multi-classification

In this section, we compare the average performance of DSCCN and other seven methods on the multi-classification of multiple breast cancer subtypes. From Table 4, we can find that DSCCN outperforms other methods across all metrics. Specifically, DSCCN achieves the highest accuracy value of 0.906 and F1-marco of 0.922, respectively. Overall, the results in Table 4 demonstrate that DSCCN is an effective method in classifying multiple breast cancer subtypes.

**Table 4:** Overall performance of all methods on multi-classification for all subtypes of breast cancer

Methods	Accuracy	Precision-macro	Recall-macro	F1-macro
Ensmble EN	0.800	0.859	0.759	0.806
Ensmble RF	0.743	0.748	0.630	0.684
Concate EN	0.800	0.859	0.759	0.806
Concate RF	0.790	0.838	0.720	0.775
DIABLO	0.604	0.589	0.632	0.609
SMPSL	0.810	0.793	0.720	0.755
DeepMo	0.849	0.884	0.820	0.851
DSCCN	**0.906**	**0.941**	**0.905**	**0.922**

The best results are marked in bold

**Fig. 3:**
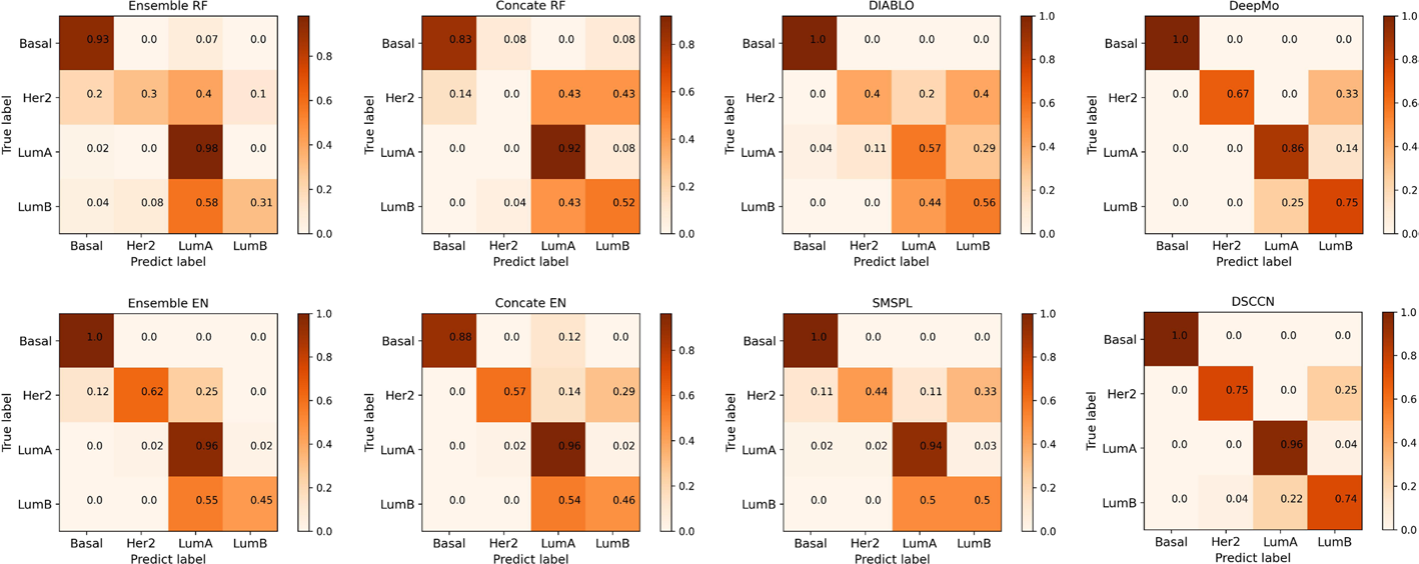
Normalized confusion matrices of all competing methods on the breast cancer multi-omics dataset. In the confusion matrix of each method, the label of each row corresponds to the true label of breast cancer subtype and the label of each column represents the predicted label of breast cancer subtype. The diagonal entity in the matrix indicates the proportion of correctly predicted classes. The off-diagonal entity in the matrix indicates the proportion of misclassification. To account for imbalanced sample sizes of different breast cancer subtype dataset, the confusion matrices are normalized within the range of 0 to 1

In Fig. 3, we plot the normalized confusion matrices to visualize the multi-classification performance of all methods for each breast cancer subtype. Figure 3 shows that DSCCN obtains comparative performance as compared to other methods on the breast cancer datasets. Specifically, for Basal, DSCCN makes accurate classifications (error rate = 0). For Her2, which has the smallest sample size, DSCCN shows the strongest classification capability (error rate = 25%) compared to other methods. For LumA, which has the largest sample size, DSCCN makes the second best classification on it (error rate = 4%). DSCCN makes a slightly weak classification of LumB (error rate = 26%). Compared to other methods, DSCCN has overall demonstrated robust performance in the classification of each subtype.

### Ablation experiment

In this section, we will evaluate the effectiveness of different parts of DSCCN by conducting ablation study on both binary classification and multi-classification. In DSCCN, two optimization techniques are employed for the classification of breast cancer subtypes, with the utilization of a DNN model as the classifier. Specifically, the first technique is to perform the differential analysis on both omics datasets to reduce data dimensionality. The other optimization technique is to detect the highly correlated genes between mRNA and DNAm using the algorithm FGL-SCCA.

As can be seen in Table 5, we construct five models for DSCCN. For DSCCN1, none of the optimization techniques is implemented. For DSCCN2, only the differential analysis is implemented. For DSCCN3, only the FGL-SCCA technique is implemented.

**Table 5:** Optimization techniques and classifier used in different DSCCN models

Models	Optimization techniques	Classifier
DSCCN1	None	DNN
DSCCN2	Differential analysis	DNN
DSCCN3	FGL-SCCA	DNN
DSCCN4	Differential analysis; FGL-SCCA	XGBoost
DSCCN5	Differential analysis; FGL-SCCA	DNN without attention mechanism
DSCCN	Differential analysis; FGL-SCCA	DNN

To investigate the efficacy of the DNN model in the classification of breast cancer subtypes, we construct two models: DSCCN 4 and DSCCN 5. For DSCCN4, two optimization techniques are employed, and XGBoost [45] is utilized as a classifier to demonstrate the effectiveness of the DNN model. To further understand the role of the attention mechanism within DNN, we construct DSCCN5, which is identical to the DSCCN except for the deactivation of the attention mechanism.We then compare the performance of these five models to explore the effectiveness of each step of DSCCN.

#### Binary classification

In this section, we discuss the performance of different modes of DSCCN on the binary classification of breast cancer subtypes. Table 6 shows the performance of classifying any two subtypes of breast cancer using different DSCCN modes depicted in Table 6. As depicted in Table 6, the indicators of DSCCN2 are superior to those of DSCCN1 on the most datasets. This implies that the differential analysis effectively filters out irrelevant feature values, resulting in the model exhibiting enhanced classification performance. Moreover, DSCCN3 outperforms DSCCN1 on all datasets in terms of ACC and AUC. This demonstrates the benefit of using FGL-SCCA to identify highly correlated features for the binary classification of breast cancer subtypes.

Moreover, Table 6 shows that the performance of DSCCN surpasses that of DSCCN4 and DSCCN5. This result further confirms that the DNN models can achieve superior results in the binary classification of breast cancer subtypes. Additionally, it demonstrates the efficacy of the attention mechanism within DNN models, significantly enhancing its performance. Overall, the ACC, AUC values, and the F1 score of DSCCN are all superior to those of its variant models. This indicates that DSCCN exhibits robust classification performance on the binary classification of breast cancer. Overall, the comparisons of different DSCCN modes demonstrate the effectiveness of combining differential analysis and Sparse Canonical Correlation Analysis to perform binary classification on breast cancer subtypes.

**Table 6:** Performance of different DSCCN models on each binary classification dataset

Breast Cancer Subtype dataset	DSCCN1	DSCCN2	DSCCN3	DSCCN4	DSCCN5	DSCCN
*Accuracy*						
Basal vs Her2	0.833	0.867	0.880	0.924	0.842	**0.926**
Basal vs LumA	0.947	0.958	0.958	0.978	0.767	**0.982**
Basal vs LumB	0.943	0.953	0.948	0.953	0.610	**0.965**
Her2 vs LumA	0.905	0.937	0.914	0.936	0.905	**0.951**
Her2 vs LumB	0.896	0.902	0.913	0.843	0.825	**0.926**
LumA vs LumB	0.695	0.768	0.766	0.786	0.635	**0.844**
*AUC*						
Basal vs Her2	0.950	0.960	**0.984**	0.973	0.957	0.982
Basal vs LumA	0.972	0.990	0.980	**0.997**	0.979	**0.997**
Basal vs LumB	0.965	0.963	0.997	0.933	0.965	**0.997**
Her2 vs LumA	0.946	0.970	0.969	0.966	0.981	**0.948**
Her2 vs LumB	0.910	0.949	0.934	0.943	0.650	**0.951**
LumA vs LumB	0.857	0.808	0.878	0.847	0.757	**0.857**
*F1-score*						
Basal vs Her2	0.742	0.769	0.833	0.822	0.686	**0.933**
Basal vs LumA	0.965	0.975	0.967	0.986	0.848	**0.988**
Basal vs LumB	0.962	0.968	0.943	0.972	0.694	**0.974**
Her2 vs LumA	0.950	0.966	0.955	0.962	0.950	**0.973**
Her2 vs LumB	0.906	0.940	0.896	0.909	0.902	**0.956**
LumA vs LumB	0.667	0.642	0.695	0.598	0.401	**0.883**

The best results are marked in bold

#### Multi-classification

In this section, we discuss the performance of different modes of DSCCN on the multi-classification of the breast cancer subtypes. Table 7 shows the performance of different DSCCN modes for classifying multiple breast cancer subtypes in Table 1. As shown in Table 7, compared to DSCCN1, the optimized DSCCN2 and DSCCN3 both demonstrate superior performance, which robustly validates the effectiveness of the two optimization techniques used. Furthermore, as shown in Table 7, the performance of DSCCN surpasses that of DSCCN4 and DSCCN5, further confirming the enhanced ability of attention mechanism-equipped DNN models in the multi-classification of breast cancer subtypes. These results suggest that a more accurate multi-classification of breast cancer subtypes can be achieved by integrating differential analysis and Sparse Canonical Correlation Analysis.

**Table 7:** Performance of different DSCCN models on multi-classification for all subtypes of breast cancer

Models	Accuracy	Precision-macro	Recall-macro	F1-macro
DSCCN1	0.783	0.605	0.614	0.609
DSCCN2	0.840	0.809	0.825	0.817
DSCCN3	0.830	0.600	0.676	0.633
DSCCN4	0.868	0.787	0.749	0.768
DSCCN5	0.774	0.581	0.570	0.575
DSCCN	**0.906**	**0.880**	**0.864**	**0.872**

The best results are marked in bold

**Fig. 4:**
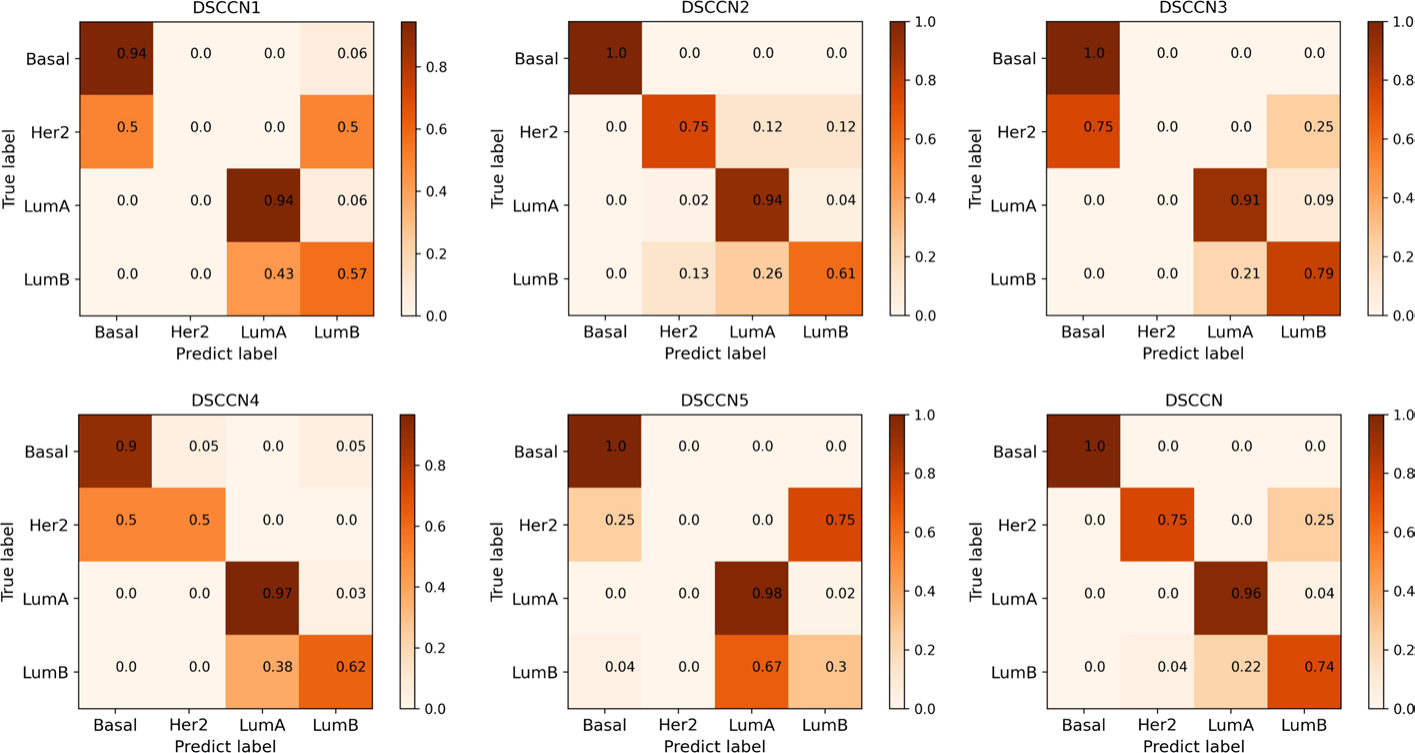
Normalized confusion matrices of different DSCCN models on the breast cancer multiclassification dataset

In Fig. 4, we generate normalized confusion matrices to visualize the multi-classification performance of each DSCCN mode on each subtype. As shown in the Fig. 4, DSCCN2, DSCCN3, DSCCN5 and DSCCN correctly classify Basal from other three breast cancer subtypes. For Her2, both DSCCN2 and DSCCN obtain the best accuracy of 75%. For LumA, DSCCN achieves the second best accuracy of 96%. For LumB, DSCCN reaches the accuracy of 74%. Overall, DSCCN consistently maintains a high classification accuracy across all subtypes, making its overall performance superior. These results highlight the significant enhancements achieved by incorporating differential analysis and FGL-SCCA techniques into our model, ensuring more reliable and precise multi-classifications on breast cancer subtypes.

### Analysis of the selected gene of DSCCN

**Fig. 5:**
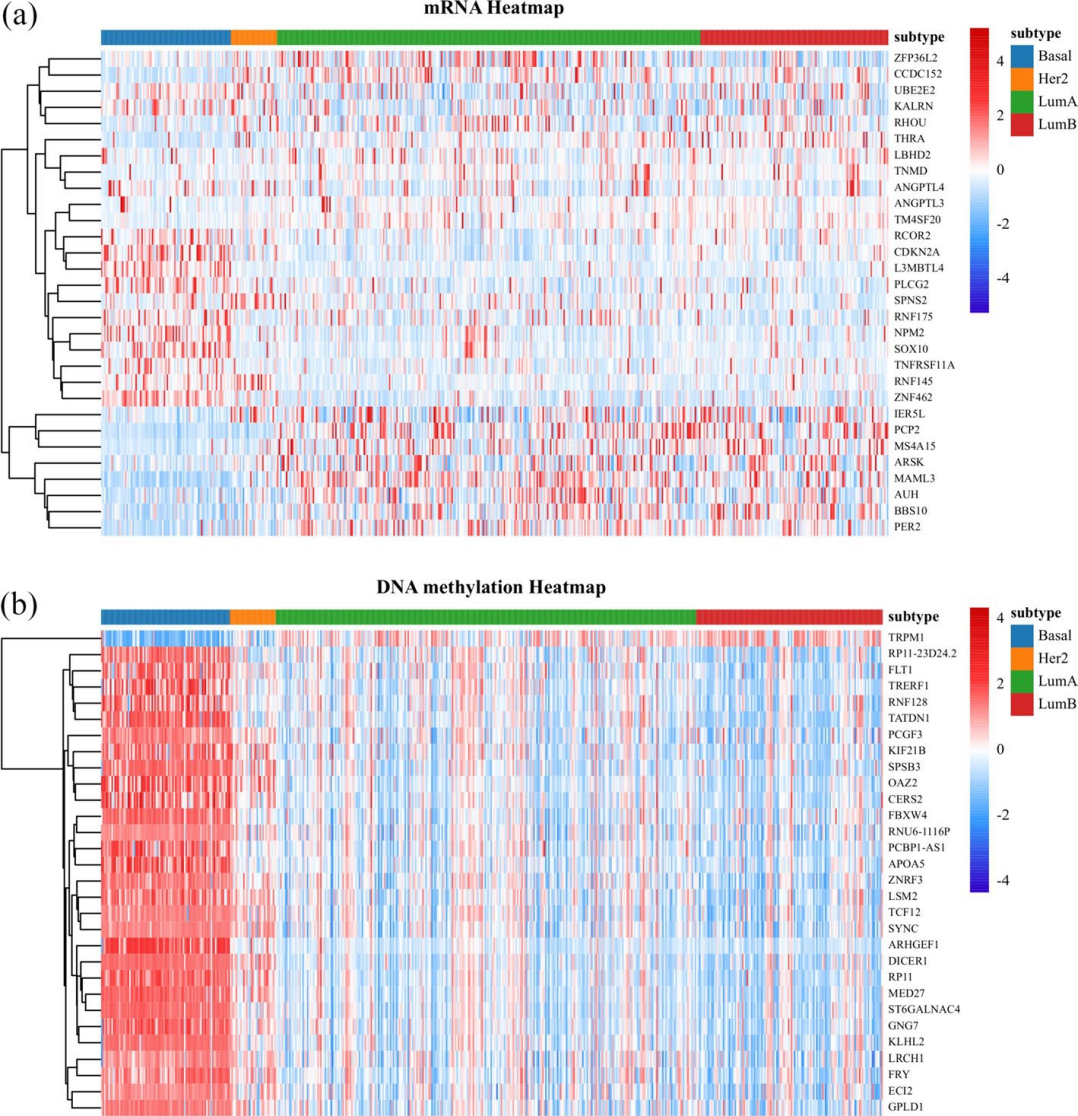
The heatmap of the expression of the top 30 selected genes of DSCCN in mRNA and DNAm omics for four breast cancer subtypes

**Fig. 6:**
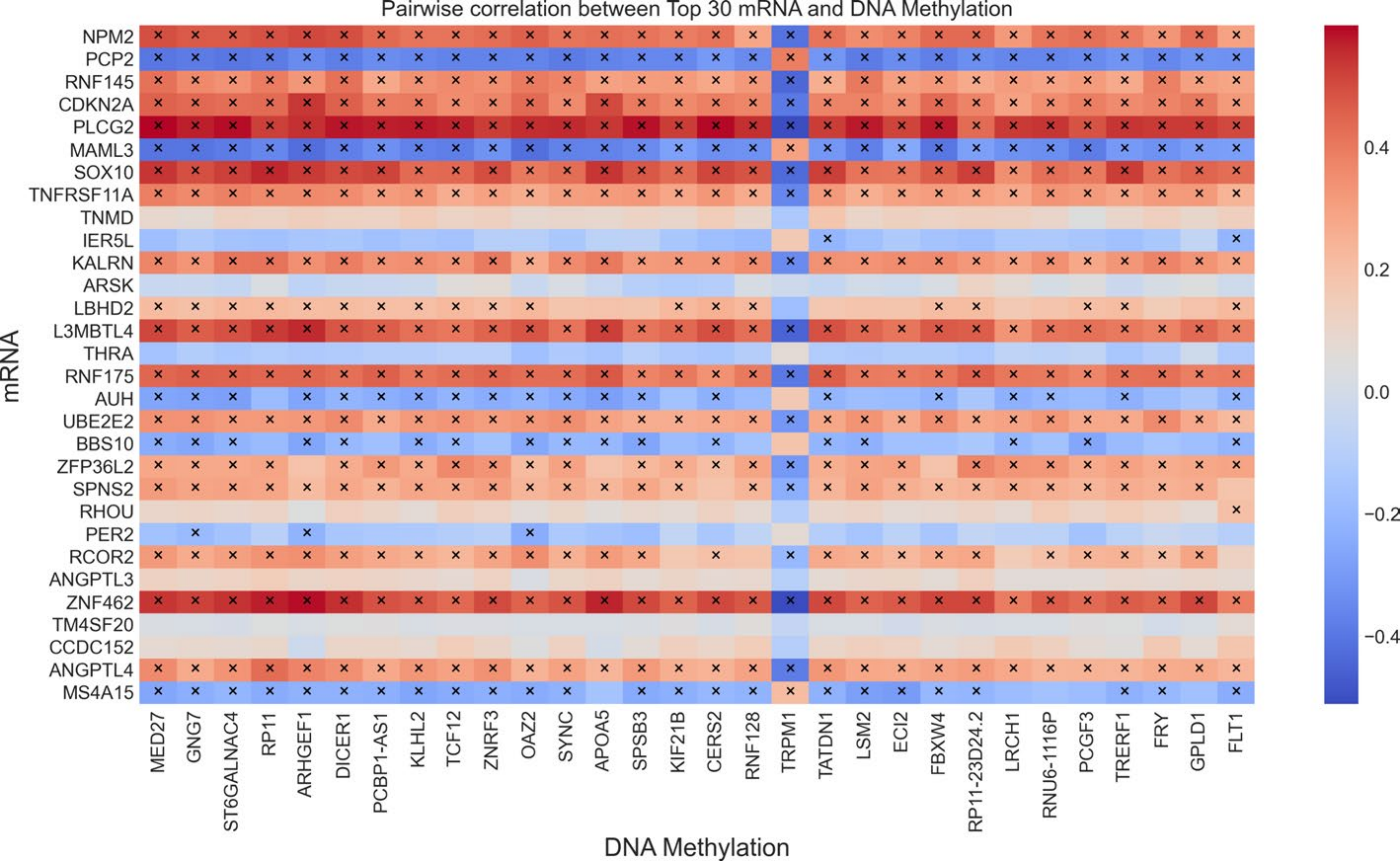
Pairwise correlation between top 30 mRNA and DNA methylaiton. Each cell represents the Pearson correlation coefficient for a specific pair of genes (i.e. NPM2 and MED27). The depth of color within each cell indicates the strength of the correlation, with a deeper color (tending towards red) indicating a higher degree of association, and ‘ × ’ denotes that the gene pair reaches the significance level (*p*-value < 0.00001)

In order to learn the differences in the expression of the selected genes in each subtype, in Fig. 5, we draw the heatmaps for the expression of the top 30 selected genes of DSCCN in mRNA and DNAm data in the multi-classification of four breast cancer subtypes. In Fig. 5, it can be observed that there exists significant expression difference in the identified genes between the Basal subtype and other subtypes. Furthermore, to investigate whether the genes detected by DSCCN are highly correlated, we selects these top 30 genes with the highest weights from each omics for Pearson correlation analysis. Figure 6 depicts the correlation coefficient matrix between gene pairs of omics, as can be seen, a significant majority of gene pairs demonstrate some correlation. Further statistical analysis reveals that 65.3% (588 of 900) of these gene pairs have *p*-values below the critical threshold, suggesting that the correlations observed among them are not due to random chance.

Interestingly, 13 out of the 30 identified mRNAs in DSCCN (RNF145, CDKN2A, PLCG2, SOX10, TNFRSF11A, L3MBTL4, THRA, BBS10, ZFP36L2, SPNS2, RHOU, PER2, ANGPTL4) have recently been found to be associated with breast cancer. For example, The CDKN2A gene was found to be a potential addition to the small list of other genes examined for associations with breast cancer histopathology and/or disease course [46]. SOX10 was recently reported to have high expression in the triple negative breast cancer, which could be helpful for diagnosing the origin of breast cancer [47]. ANGPLT4 has been identified to be associated with the malignant progression and poor prognosis of breast cancer. This implies that ANGPLT4 might serve as a novel therapeutic target for breast cancer [48].

18 out of the 30 identified DNAms in DSCCN (MED27,GNG7, ST6GLNAC4,RP11,DICER1, TCF12, ZNRF3, APOA5, CERS2,TRPM1,TATDN1,LSM2,ECI2,FBXW4,TRERF1,FRY,GPLD1,FLT1) have been confirmed to be associated with breast cancer. For instance, the expression level of MED27 in breast cancer samples is higher than in normal tissues, especially in triple-negative breast cancer.Additionally, as the pathological stage increases, its expression level also rises [49]. The study revealed that, compared to normal breast tissue, GNG7 exhibits lower expression in breast cancer tissue. Silencing GNG7 significantly enhances cell proliferation, inhibits apoptosis, and the exogenous overexpression of GNG7 has a reversing effect on breast cancer cells [50].

## Conclusion

In this work, we present a method called DSCCN to classify breast cancer subtypes using multi-omics data. To address the challenges of large *p* small *n* issue and data heterogeneity problem in multi-omics data integration, we first perform differential analysis on the multi-omics expression data of patients to identify differentially expressed genes and obtain DE-mRNA features and DE-DNAm features. Then we carry out Sparse Canonical Correlation Analysis to identify highly correlated DE-mRNA and DE-DNAm features. Finally, we adopt a neural network with attention mechanism to identify genes with high cosine similarity to classify breast cancer subtypes. Through the use of Sparse Canonical Correlation Analysis and attention mechanism, DSCCN is able to efficiently identify highly correlated genes between mRNA and DNAm data. The experimental results show that our proposed method is superior to the existing methods in the binary classification and multi-classification of breast cancer subtypes. The ablation study shows that each step of DSCCN has a significant contribution to the classification performance. DSCCN thus could be a useful framework for classifying breast cancer subtypes.

Despite the effectiveness of DSCCN in classifying breast cancer subtypes, limitations remain. Biological intuition says that using more omics data could improve the performance of the classification model. It is known that mRNA and DNAm are typical coding genes. In the future, we intend to extend our analysis to non-coding genes, especially the analysis of miRNAs and lncRNAs. This may enable us to improve the classification accuracy and robustness of our model and understand the breast cancer subtypes from a comprehensive perspective of coding and non-coding genes. Moreover, due to data imbalance in breast cancer dataset, our model is difficuilt to thoroughly learn the features of each subtype, which results in a decreased accuracy. Considering that data augmentation techniques have been proven effective in numerous fields, we intend to incorporate these techniques into our future work so as to accurately recognize the characteristics of each subtype.

## Data Availability

The source code and data are available at https://github.com/hyr0771/DSCCN.
